# Operative vaginal delivery: Number 7 – July 2023

**DOI:** 10.1055/s-0043-1772581

**Published:** 2023-08-18

**Authors:** Álvaro Luiz Lage Alves, Lucas Barbosa da Silva, Breno José Acauan Filho, Rodrigo Dias Nunes

**Affiliations:** 1 Hospital das Clínicas, Universidade Federal de Minas Gerais, Belo Horizonte, MG, Brazil.; 2 Hospital das Clínicas, São Sebastião, SP, Brazil.; 3 Escola de Medicina da Pontifícia Universidade Católica do Rio Grande do Sul, Porto Alegre, RS, Brazil.; 4 Universidade do Sul de Santa Catarina, Palhoça, SC, Brazil.

## Key points

- When the correct technique is applied, forceps and vacuum extractors have low rates of complications.
- For the fetus with signs of hypoxia in the expulsive phase, operative vaginal delivery has the potential to reduce exposure to intrapartum factors that promote hypoxic-ischemic encephalopathy.
- Medium and/or rotational forceps are appropriate options in selected circumstances and require skill and experience.
- Even though forceps are more effective than vacuum extraction for operative vaginal delivery, they are more associated with severe perineal lacerations.
- Cephalohematoma is more likely to occur with increasing duration of vacuum extraction.
- Flexible vacuum cups have higher failure rates, but lower incidences of trauma to the newborn’s scalp.

## Recommendations

- Operative vaginal delivery is contraindicated if the fetal head is not engaged, delivery presentation is unknown, or if the fetus has suspected or diagnosed bone demineralization or bleeding disorders.
- Ultrasound evaluation prior to instrumentation of labor is recommended when there is doubt in the clinical assessment of delivery presentation.
- Routine episiotomy is not recommended in operative vaginal delivery because of poor healing and discomfort associated with mediolateral episiotomy, and the risk of injury to the anal sphincter and rectum with midline episiotomy. When individually indicated, it should be mediolateral and performed only after a successful traction test.
- In the prolonged pelvic period of fetuses estimated to weigh more than 4,500 grams, intrapartum cesarean section for prevention of shoulder dystocia is preferable to low operative vaginal delivery or outlet delivery. Similarly, operative vaginal delivery with the fetal head in the mid pelvis should be avoided in fetuses estimated to weigh more than 4,000 grams, and intrapartum cesarean section is indicated. In these situations, instrumental delivery should only be considered in the presence of experienced operators, through individual assessment of fetal position and size, history of previous deliveries and maternal habits.
- The attempt to use forceps must be interrupted if there is no progression of the cephalic pole after three tractions performed with correct grip by an experienced operator.
- Vacuum extraction should be avoided before 32 weeks and caution should be exercised between 32 and 36 weeks, as the lower safe limit for gestational age has not been established yet.
- Vacuum extraction should be stopped when there is no evidence of progressive descent of the fetal head or when the cup detaches on three occasions.
- Sequential use of vacuum extraction and forceps is associated with increased neonatal complications and should not be routinely performed. After a failed vacuum extraction attempt, the risks and benefits of a sequential attempt to use a forceps or cesarean section should be evaluated.
- Neonatologists must be informed about the technique used in operative vaginal delivery.

## Background

Operative vaginal delivery is used to provide a safe birth via the vaginal route based on maternal and fetal indications. Its greater benefits are the prevention of a cesarean section and its associated morbidities, as well as neonatal complications arising from intrapartum hypoxia. 1

Although the forceps has been presented as the resource with the greatest potential for saving lives in the history of medicine, its current replacement by cesarean section is a result of the lack of preparation of the new generation of obstetricians, the inability of professors to teach its practice and the growing medical judicialization of obstetrics. The forceps instrument currently holds stigma and social prejudice arising from maternal and neonatal trauma caused by misuse. Vacuum extractors are more contemporary instruments, and although less effective than forceps, they are easier to use and have advantages that have made them instruments of choice in several countries. 2

In recent decades, an increase in the rates of cesarean sections performed in the second stage of labor has been observed with a concomitant reduction in operative vaginal delivery. Difficult fetal extraction in cesarean section is an event associated with failure or lack of attempt at operative vaginal delivery, potentially aggravating maternal and neonatal morbidity. Therefore, the acquisition of skills and competences related to the use of forceps and vacuum extractors has become essential in the current process of training obstetricians. 3

## What are the main indications and contraindications for operative vaginal delivery?

For the fetus with signs of hypoxia in the expulsive phase, operative vaginal delivery has the potential to reduce exposure to intrapartum factors that promote hypoxic-ischemic encephalopathy. 1 The main indications for operative vaginal delivery are signs of acute fetal hypoxia, maternal exhaustion, prolonged expulsive period, umbilical cord prolapse with complete cervical dilation, sudden death of the parturient, arrested labor, persistent asynclitism, rotational dystocia, third-degree deflected cephalic presentation (face) with variety of anterior chin position, resistance of the soft tissue, uterine inertia, poor abdominal press. The aim of forceps (or vacuum) called prophylactic (relief) is to reduce the effort and discomfort of the pelvic period. Operative delivery is useful in maternal conditions or complications that contraindicate expulsive effort (cardiopathies, severe respiratory diseases, stroke, aneurysm, esophageal varices, spinal cord trauma, myasthenia gravis, proliferative retinopathy, neuromuscular pathologies, etc.), in preventing the non-reassuring fetal status and in pelvic vaginal delivery when the head is stuck after failure of the initial maneuvers. 4 5

Because it causes less maternal trauma than the forceps, the vacuum extractor is an excellent alternative for operative vaginal delivery, especially for outlet delivery. Its indications are similar to those of the forceps. However, as the vacuum extractor requires more time for fetal extraction, it should not be the preferred method in emergency situations. The main advantages of vacuum extraction include a reduction in application errors, greater ease of learning, the possibility of self-direction and autorotation, less use of force on the fetal head, less need for analgesia and episiotomy and the reduction of birth canal lacerations. Vacuum-extractors with flexible cups cause less severe trauma to the fetal scalp than those with rigid cups, and should be preferred in simple vaginal deliveries. 4 5

Operative vaginal delivery is contraindicated if the fetal head is not engaged or the delivery presentation is unknown. The following are absolute contraindications to operative vaginal delivery: cephalopelvic disproportion, total or partial placenta previa and anomalous presentations such as transverse, second-degree deflected cephalic (forehead) and third-degree deflected cephalic (face) with a variety of posterior chin positions. It is also relatively contraindicated if the fetus has suspected or diagnosed bone demineralization (osteogenesis imperfecta) or bleeding disorders (hemophilia, Von Willebrand disease, alloimmune thrombocytopenia). Operative vaginal delivery in fetuses weighing more than 4,000 grams must be judicious when choosing either forceps or the vacuum extractor. With regard to fetuses with an estimated weight of less than 2,000 grams, forceps are the safest instrument and can be used in fetuses as small as 1,000 grams. 4 5

In the prolonged pelvic period of fetuses estimated to weigh more than 4,500 grams, intrapartum cesarean section to prevent shoulder dystocia is preferable to low operative vaginal delivery or outlet delivery. Similarly, operative vaginal delivery with the fetal head in the mid pelvis (De Lee station 0 and + 1) should be avoided in fetuses weighing more than 4,000 grams, and intrapartum cesarean section is indicated. In these situations, instrumental delivery should only be considered in the presence of experienced operators, through individual assessment of fetal position and size, history of previous deliveries and maternal habits. 6

Vacuum extraction is not risk free (cerebral and retinal hemorrhage), and is also contraindicated in prematurity (gestational age < 32 weeks). Between 32 and 36 weeks, the vacuum extractor must be used with great caution, as the lower safety limit for gestational age has not been established yet. As the fetal extraction time with the vacuum extractor is prolonged, the instrument should also not be used if there are signs of fetal hypoxia. Vacuum extractors are also not indicated for pelvic vaginal delivery (breech baby) nor for face presentation, and should be replaced by forceps in these situations. Contraindications to vacuum extraction, although relative, also include: previous collection of blood or trauma to the fetal scalp, fetal death, anomalies of the cephalic pole (anencephaly, hydrocephalus), macrosomia and negative test traction in a previous attempt to use forceps. 5 7

## What are the main instruments currently recommended for operative vaginal delivery?

Forceps and vacuum extractors are the main instruments recommended for extracting the fetus from the birth canal, performed by grasping and pulling the fetal cephalic pole. The choice of instrument is related to the operator’s preference and experience, and to maternal and fetal conditions. 8 9

Forceps are instruments with two broad branches, each with four components: blade (seizes the cephalic pole), shank (or pedicle; located between the handle and the blade), joint and handle. The best known models nowadays are Simpson, Kielland, Piper and Marelli. 9

Although forceps are more effective than vacuum extractors, they are more associated with severe perineal lacerations. Simpson’s forceps are the most widespread worldwide. It features crossed branches, English (by fitting) fixed lock, handle with finger grips and fins (finger support) and fenestrated blades. The cephalic (adapts to the cephalic pole) and pelvic (adapts to the maternal pelvis) curvatures of the blades are prominent, and this specificity is advantageous for the grip and traction of the cephalic pole. It has three sizes, with shank lengths of 30, 33 and 35 cm. 4 5 9

**Figure 1.**
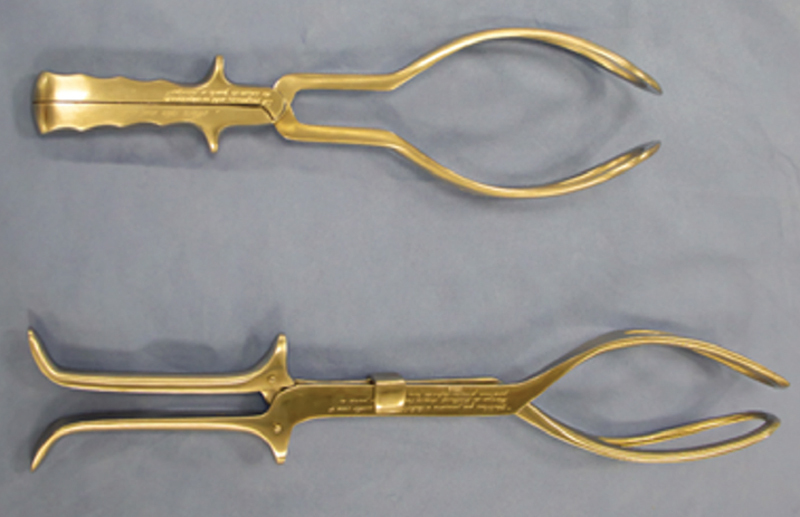
Source: photographic record by the authors. Simpson (upper) and Kielland (lower) forceps

Kielland’s forceps have crossed branches, but the articulation is performed by sliding, allowing the asymmetrical application of the blades in the vagina and the correction of asynclitism. It is 39cm long, handles are smooth with fin and identification buttons (knobs) on the front side. In the articulated instrument, the shanks are superimposed with the right above the left. Blades are fenestrated with smooth and rounded edges, and have very discreet cephalic and pelvic curvatures, which makes it an specific instrument for wide rotations ( Figure 1 ). 9

**Figure 2.**
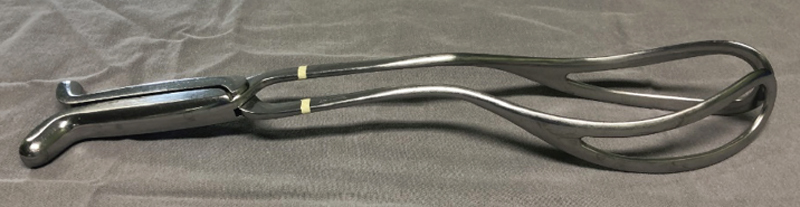
Source: photographic record by the authors. Piper’s forceps

The Piper’s forceps are specific instruments for extracting the head (breech baby) in pelvic delivery. It has long (44cm long) crossed branches, English lock and handle without finger grips and fins. Blades are fenestrated with very prominent cephalic and pelvic curvatures. A third curvature, the perineal, is present on the underside of the handles, close to the blades ( Figure 2 ). 9

**Figure 3.**
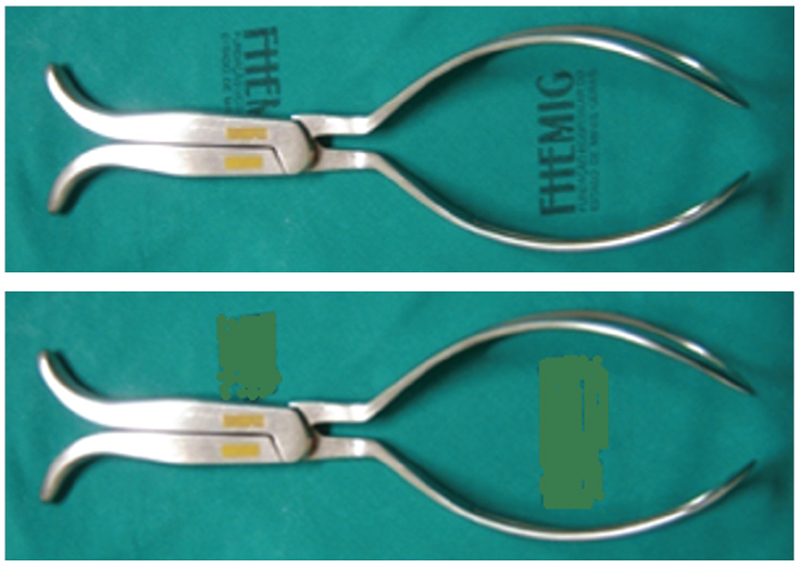
Source: photographic record by the authors. Marelli’s forceps

Marelli’s forceps are specific for fetal extraction in cesarean sections. It has crossed branches, English lock and smooth handle without fins. Its blades are fenestrated without a pelvic curvature (“bayonet” shaped blade), since fetal extractions with this instrument are performed through the abdomen ( Figure 3 ). 5 9

**Figure 4.**
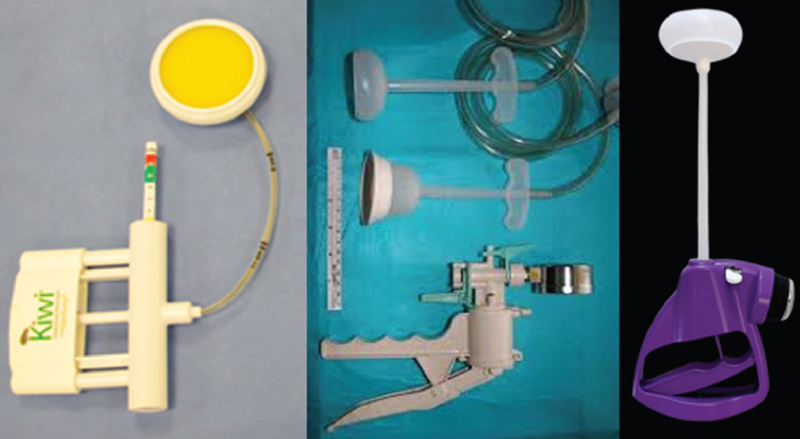
Sources: photographic records by the authors; https://www.panamedical.com.br/vacuo-extratores . Kiwi Omni Cup® (left), Mityvac® (center) and Mystic II (right) vacuum extractors

Vacuum extractors are instruments that have a cup, a connecting tube and a suction pump. By means of negative pressure, the cup, applied to the scalp, pulls the fetal head. Cups can be rigid (made of metal), semi-rigid or flexible and have a bell or mushroom shape ( Figure 4 ). Flexible bell vacuum extractors have higher failure rates, but lower incidences of trauma to the newborn’s scalp. 8

**Figure 5.**
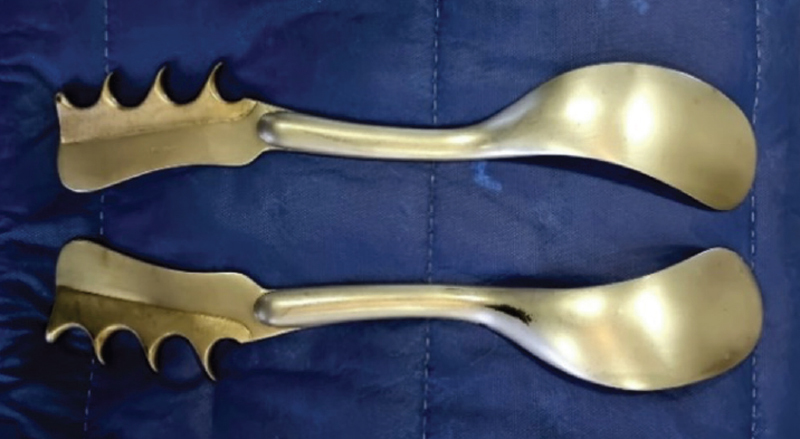
Source: photographic record by the authors. Thierry’s spatulas

Spatulas and the Odon device are less widespread instruments. Spatulas are instruments with two independent and symmetrical branches that do not articulate. Each branch has a stem, handle and solid, wide blade. The branches act as independent levers and the fetal head is not pinched between the blades. The action of the spatulas is similar to that of the shoe presser, whose function is to help slide. Thierry, Velasco and Teissier spatulas are described. 10 Velasco’s spatulas are smaller and straighter. Thierry’s spatulas are larger and have a slight pelvic curvature at the upper edge of the blade ( Figure 5 ). Compared to forceps and vacuum extractors, neonatal complication rates for spatulas appear to be similar or lower. Rates of severe perineal lacerations are also similar, but vaginal wall lacerations are more common. 11

**Figure 6.**
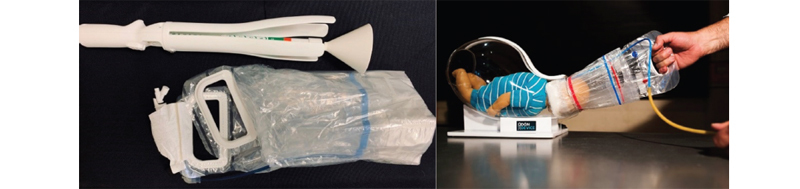
Source: Adapted from Odon Device (2020) 12 and Silvestri (2013) 13 . Odon device

The Odon device is a film-type polyethylene instrument that creates an air envelope around the fetal head, allowing extraction by means of traction ( Figure 6 ). 12 13 It has the potential to be safer and easier to apply than forceps and vacuum extractors. Currently, it is being used in multicenter experimental clinical trials, although not yet cleared by regulatory agencies for clinical practice. In a pilot observational study, the success rate at birth was close to 50% without severe maternal or neonatal adverse outcomes, but lower than those of the other instruments. 14

## How should operations in operative vaginal delivery be classified?

Classifications of operations in operative vaginal delivery are based on pelvic planes and delivery mechanisms. The application performed before the engagement of the cephalic pole (“high forceps”) is contraindicated. The American College of Gynecology and Obstetrics (2015), endorsed by the Royal College of Obstetricians and Gynecologists (2020) has the most current classification ( Chart 1 ). 4 5

**Chart 1.** American College of Gynecology and Obstetrics Classification of Operative Vaginal Delivery (2015) 4

Type	Findings
Outlet	The fetal scalp is visible at the vaginal introitus without separating the labia minora; the fetal skull has reached the pelvic floor and is near or occupying the perineum; the sagittal suture is in the antero-posterior (OA, OP) or oblique (LOA, ROA, LOP, LOP) diameter, with rotation not exceeding 45°.
Low	Cephalic apex in the De Lee plane + 2 or below, without reaching the pelvic floor. Two situations may occur: a) Rotation ≤ 45° (LOA, ROA, LOP, ROP); b) Rotation > 45° (include LOT and ROT).
Mid	The cephalic pole is engaged, but above De Lee’s plane + 2; rotation can be ≤ 45° or > 45°.

OA: occiput anterior; OP: occiput posterior; LOA: left occiput anterior; ROA: right occiput anterior; LOP: left occiput posterior; ODP: occipito-right-posterior; LOT: left occiput transverse; ROT; right occiput transverse

## What are the prerequisites for performing an operative vaginal delivery?

The main prerequisites for operative vaginal delivery include information and agreement on the benefits and risks of the procedure, adequate maternal pelvis, fetal weight estimate performed (clinical or ultrasound), engagement of the cephalic pole, complete cervical dilation and effacement, ruptured membranes, previous bladder emptying, knowledge of the presentation and variety of position, and satisfactory anesthesia (regional block in medium / rotational applications, pudendal or perineal blocks in low and outlet applications). 15

## What are the main operative times and technical details of forceps application?

The application of the forceps must be preceded by a urinary catheter and satisfactory maternal anesthesia. Low spinal anesthesia (“saddle”) is preferred, especially in emergency situations and in mid and rotational forceps. It has the advantages of quick installation, providing anesthetic blockade of the sacral fibers and perineal relaxation without interfering in uterine contractility, abdominal press and quality of pushing. In situations where the parturient is already under analgesia by epidural block, with a catheter installed, the infusion of higher doses of anesthetics will be necessary and the time for achieving satisfactory analgesia will be longer. 16

The operative times are sequentially: presentation of the instrument in front of the vulva, introduction and application, gripping of the cephalic pole, assessment of grip, traction test and definitive traction (with or without rotation). 4 5

**Figure 7.**
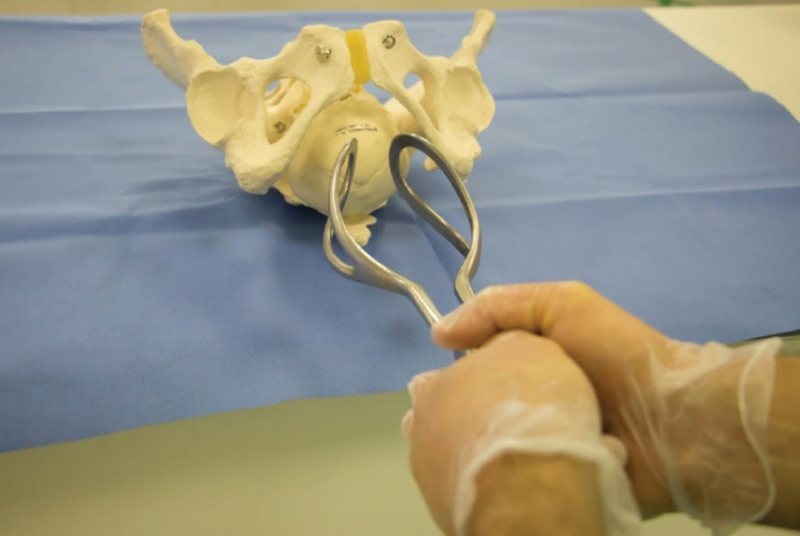
Source: photographic record by the authors. Simpson’s forceps presentation in the occiput posterior position

**Figure 8.**
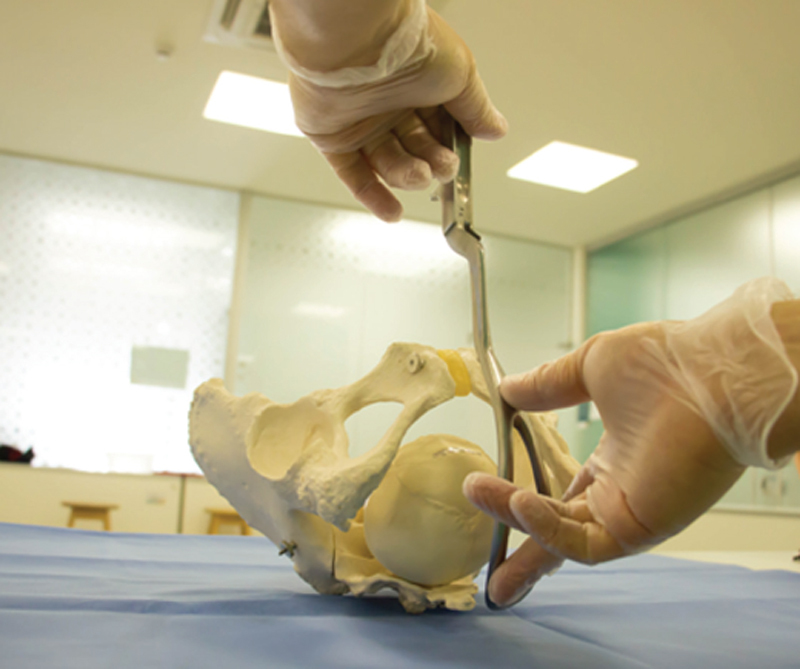
Source: photographic record by the authors. Application of the left branch of Simpson’s forceps in the occiput posterior position

**Figure 9.**
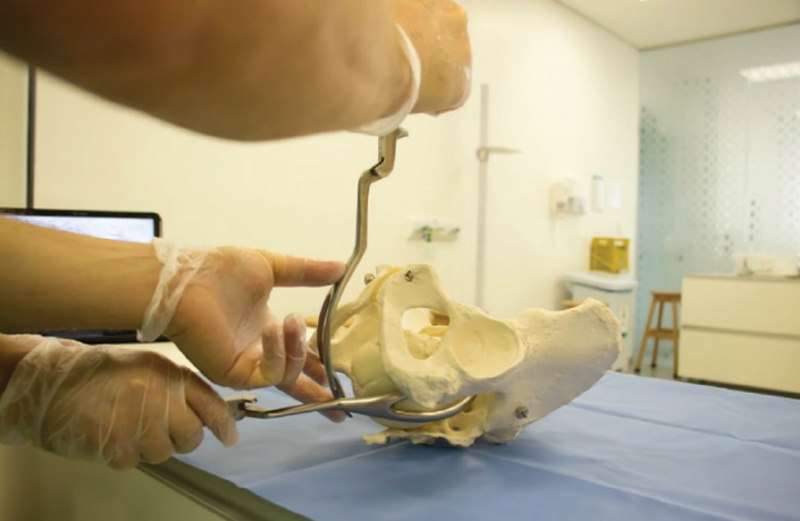
Source: photographic record by the authors. Application of the right branch of Simpson’s forceps in the occiput posterior position

**Figure 10.**
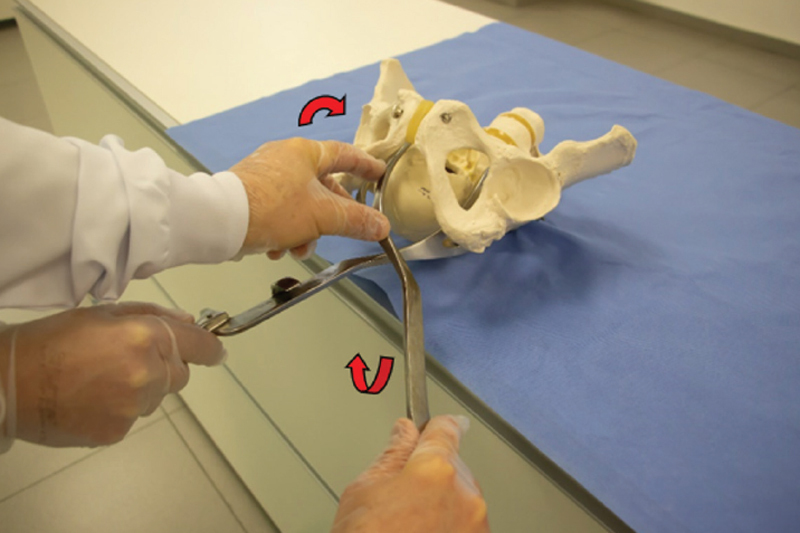
Source: photographic record by the authors. Application of the right branch of Kielland’s forceps with the La Chapelle spiral, in the left occiput anterior (LOA) position

The first stage involves presenting the instrument to the vulva, simulating the way it will look after being applied to the fetal head ( Figure 7 ). The grip includes the application (introduction and placement) and the actual grip. In the case of forceps, to apply the branches, movements of “lower introduction” are performed, always penetrating with the blades through the sacral voids (bilateral spaces between the sacrum and the hamstrings). In oblique varieties, the posterior branch must always be the first one to be applied. In transverse varieties (Kielland’s forceps), the first branch to be inserted is optional, but the anterior branch is usually preferred. In the direct varieties (occiput posterior [OP] and occiput anterior [OA]), the left branch must be applied first in order to avoid the need to uncross the branches after applying the second (right branch) ( Figures 8 and 9 ). In the rotated cephalic pole, the branch that will be applied to the anterior parietal is introduced through the triple spiral movement, which sequentially includes translation, lowering and torsion of the handle (Lachapelle’s maneuver) ( Figure 10 ). It is important to point out that manual rotation is an alternative for correcting the rotated cephalic pole (transverse and oblique position varieties). The cephalic pole is grasped with the tips of the fingers positioned on the parietal bones (thumb on one side and the other fingers on the other). During uterine contraction, the fetal head is slightly elevated, flexed and rotated, until it is positioned in a variety of OP positions. 4 5

**Figure 11.**
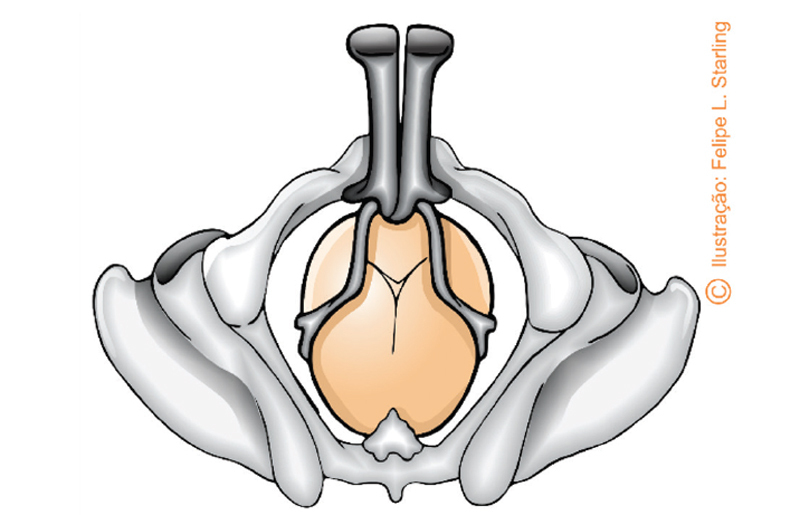
Source: Illustration by Felipe Lage Starling (authorized). Fundamental diagnostic criteria for ideal grip (Laufe)

The biparietal-mentonian is the ideal grip. Three fundamental diagnostic criteria (Laufe’s criteria) are used to check the correct grip: the small fontanel must be a transverse finger width from the plane of the handles (“in the center of the figure”); the sagittal suture must be placed perpendicularly and equidistant from the plane of the handles; the blade fenestrae should not be perceived by more than a finger pad between the grasped head and the forceps on either side ( Figure 11 ). After checking the ideal grip, the branches must be moved towards the occiput. 4 5

**Figure 12.**
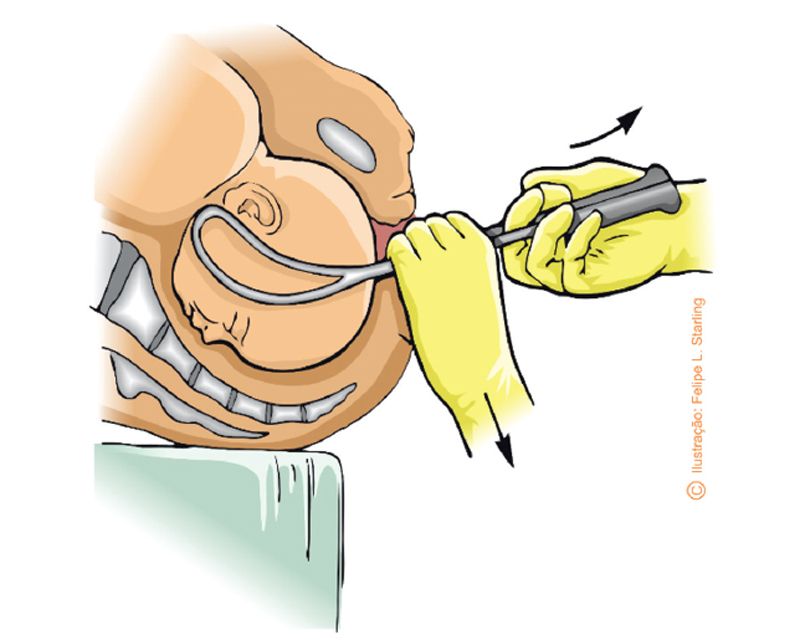
Source: Illustration by Felipe Lage Starling (authorized). Axial traction (Saxtorph-Pajot maneuver) in the occiput posterior position

The traction must be simultaneous to the contractions and performed axially, that is, in the axis of the birth canal, perpendicularly to the presentation stop plane. The operator should be seated at an adequate height with the chest at the same level as the birth canal and the arms flexed just below the table. The force must be exerted only with the arms. To obtain axial traction, the dominant hand positioned on the handles exerts force directed at the operator’s chest. Simultaneously, the other hand positioned on the rods applies downward force against the maternal perineum (Saxtorph-Pajot maneuver) by providing a 45° vector and effective axial traction ( Figure 12 ). 4 5

**Figure 13.**
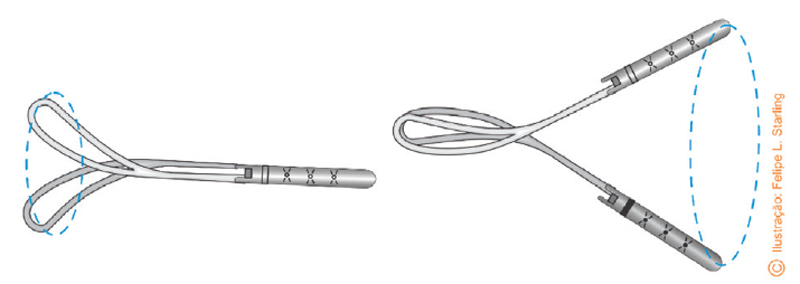
Source: Illustration by Felipe Lage Starling (authorized). Key through the keyhole rotation with Kielland’s forceps and wide circumduction movement of the handles with Simpson’s forceps

Rotation is performed in the oblique and transverse varieties simultaneously with traction. Rotation with Simpson’s forceps should be performed with a wide movement of the handles in an arc (circumduction). With the Kielland’s forceps, the movement of the handles is performed in a “key through the keyhole” movement and the rotation can be completed before traction ( Figure 13 ). Note that Simpson’s forceps are more suitable for small rotations. The Kielland’s forceps should be the instrument of choice for rotations, especially when above 45°. Once rotation is completed and successful traction is confirmed (positive traction test), with the cephalic pole with the occiput below the pubic symphysis, the need for episiotomy is assessed. 4 5

**Figure 14.**
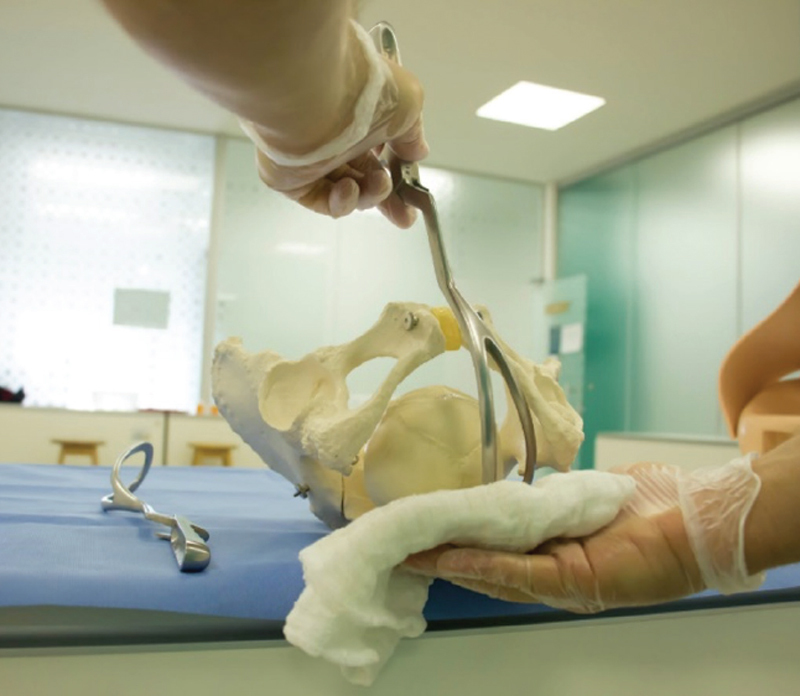
Source: photographic record by the authors. Removal of Simpson’s forceps branches in the occiput posterior position

The removal of forceps branches must precede the complete exit of the fetal head and must be performed as soon as the mandible is accessible. The branches are removed in reverse order of their application ( Figure 14 ). Detachment of the cephalic pole is completed by the modified Ritgen maneuver. After the fetal extraction and delivery are completed, the birth canal is revised and if necessary, lacerations are repaired and/or episiorrhaphy is performed. 4 5 Despite the high effectiveness for resolution of the delivery, the attempt to use forceps should be interrupted if there is no progression of the cephalic pole after three tractions performed with correct grip by an experienced operator. 4 5

## What are the main operative times and technical details of applying the vacuum extractor?

**Figure 15.**
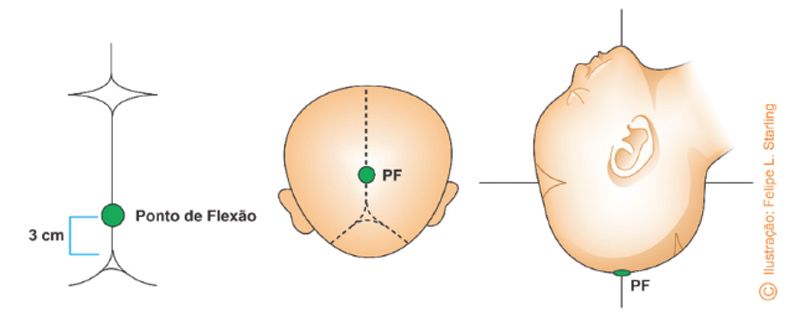
Source: Illustration by Felipe Lage Starling (authorized). Fetal cephalic pole flexion point

Pudendal nerve block may be preferable to neuraxial anesthesia when choosing vacuum extraction. Local anesthetic infiltration is performed bilaterally below the sciatic spines. Unlike forceps blades, vacuum extractor cups do not come into significant contact with the vaginal walls and do not increase the diameter of the cephalic pole. 5 15 The vacuum extractor must be tested by the operator immediately before use by creating vacuum through compression of the cup on the palm of the hand. The instrument must be presented in front of the vulva, demonstrating how the cup will be applied to the fetal head. 17 18 The fetal scalp must be dried before the cup is applied. The cup will perform the action of gripping the cephalic pole, and must be introduced in the vulvar vestibule and applied over the sagittal suture, equidistant from the parietal bones with its center 3cm in front of the lambda (at the point of flexion). With the center of the cup positioned at the flexion point, its posterior edge will be 1cm (one finger) away from the lambda ( Figure 15 ). The cup must not be inadvertently applied over the fontanels. The positioning of the cup is the same for any variety of position. In oblique position varieties (left occiput anterior [LOA], left occiput posterior [LOP], right occiput anterior [ROA], right occiput posterior [ROP]), the cup traction performed during the vacuum-extraction process promotes the descent of the cephalic pole with autorotation. 17 18

A good grasp should be checked before traction, confirming the absence of maternal tissue between the cup and the fetal head. The manometer should be calibrated to a maximum of 500 mmHg (between 350 and 500 mmHg) during contractions with a reduction to 100 mmHg during uterine relaxation. 17 18 However, maintaining pressure between 350 and 500 mmHg between contractions with the aim to avoid discontinuing the descent and detachment of the cup does not seem to increase neonatal complications and has also been recommended. 19

**Figure 16.**
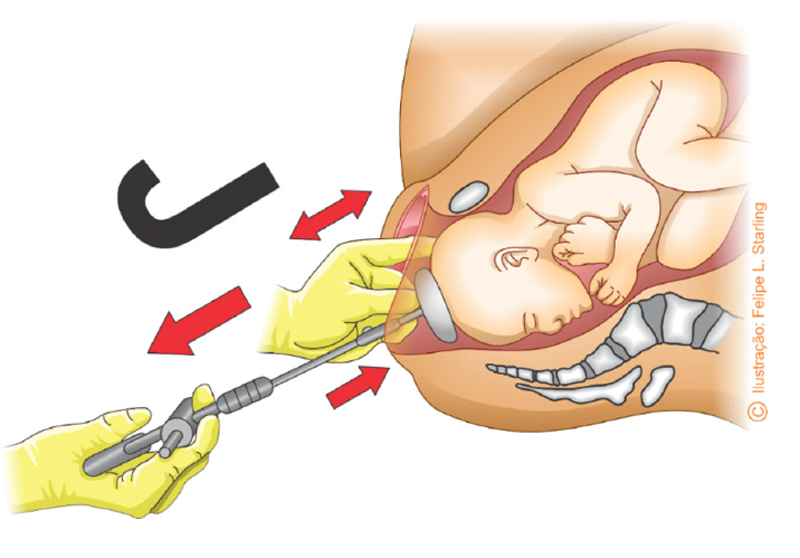
Larger red arrow: perpendicular pull force downwardsSmaller red arrow: perpendicular force maintaining the cup at the fetal cephalic pole (thumb finger) upwardsDouble red arrow: maintenance of the cup attached to the scalp (index and middle fingers)Black letter J: direction resulting from the traction in the shape of a J (Pajot’s maneuver)Source: Illustration by Felipe Lage Starling (authorized). Vacuum extraction traction technique

**Figure 17.**
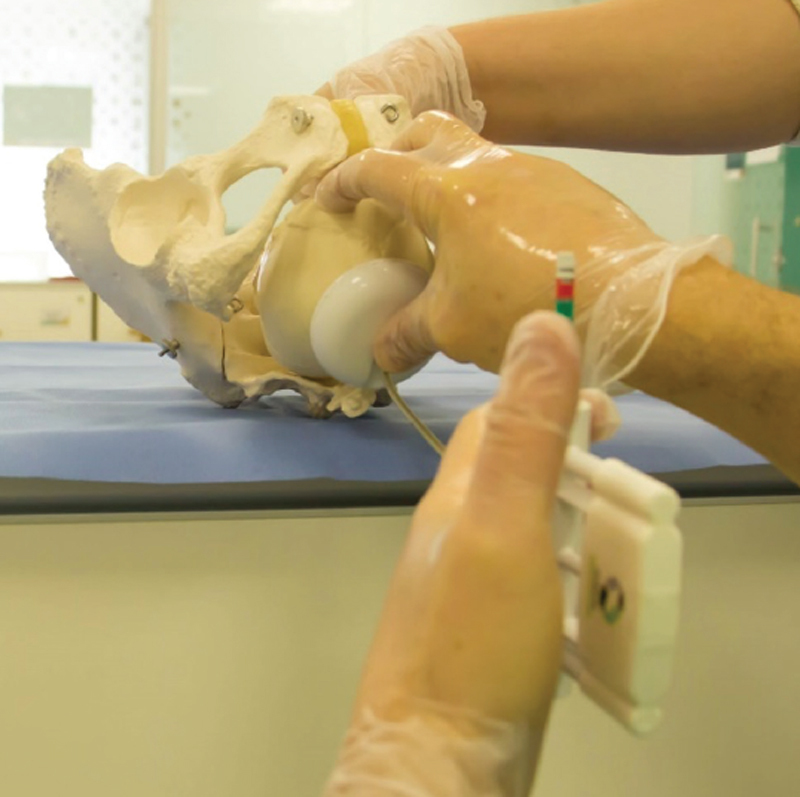
Source: Photographic record by the authors. Vacuum extraction traction technique

The operator, seated in front of the delivery table with the chest at the level of the birth canal, must pull perpendicularly to the cup plane until the occiput is positioned below the pubic symphysis. Traction performed during uterine contraction should follow the pelvic curvature (Pajot’s manuver), keeping the traction shank always straight at a 90° angle with the cup. Thus, the pulling hand exerts a perpendicular force to the planes of the cup and the fetal cephalic pole, towards the operator’s chest. Efficient traction is obtained by the imbalance between the hand that pulls and the hand that keeps the cup attached to the fetal cephalic pole, similar to a “tug of war”. This force is opposite and slightly stronger than the force exerted by the hand that keeps the cup attached to the fetal cephalic pole. The cup is kept attached to the fetal cephalic pole by means of a force that is also perpendicular and exerted in a superior direction, in the opposite direction to the traction force with a slightly weaker intensity than this, sufficient to prevent the cup from detaching during the entire action of traction. The superior steering force is exerted by the thumb positioned in the center of the cup. Simultaneously, index and middle fingers are positioned directly on the cephalic pole, thereby contributing to maintain the cup attached to the fetal scalp ( Figures 16 and 17 ). The manometer must be observed throughout the traction process in order to detect the loss of vacuum, indicative of calibration correction. 17 18

As soon as the occiput reaches the pubic symphysis, the suction pump and the connecting tube of the vacuum extractor are elevated and the need for episiotomy is assessed. After vulvar exteriorization of the fetal mandible, the cup is removed by pressing the pressure relief valve (vacuum). The extraction of the fetal cephalic pole is completed with the modified Ritgen maneuver. 17 18 Vacuum extraction is usually achieved with up to three pulls. Three additional gentle pulls are acceptable to complete the cephalic pole deflection. The vacuum extraction attempt should be stopped when there is no evidence of progressive descent of the fetal head, when the cup detaches on three occasions or when the traction time exceeds 20 minutes. During traction, the sudden detachment of the cup by loss of vacuum and vigorous movements must be avoided, as it leads to scalp lacerations. The sequential use of the vacuum extractor and the forceps is associated with increased neonatal complications and should not be routinely performed. Therefore, after a failed vacuum extraction attempt, the risks and benefits of a sequential attempt at forceps or a cesarean section must be carefully evaluated. 17 18

## What are the specific forceps techniques that require greater skill and competence by the operator?

Medium and/or rotational forceps are appropriate options in selected circumstances and require operator skill and experience. 4 5 20 The posterior oblique and transverse position varieties and the head stuck (breech baby) in pelvic delivery determine specific forceps application techniques. 9 20

In forceps in posterior oblique varieties (ROP and LOP), there are three technical options related to the model, forceps availability, and operator skill and preference. Although rotation to OP requires more skill, it should be preferred whenever possible, avoiding detachment of the cephalic pole in OA. In all application possibilities, the posterior branch must be introduced first. Subsequently, the second (anterior) branch is introduced through the Lachapelle’s maneuver. 9 20

One option is to rotate 45° in the posterior direction for OA. In this situation, the branches of the forceps are applied with the pelvic curvature of the blades in an anterior direction. Although rotation is not wide, detachment of the cephalic pole occurs in the posterior variety (OA), which requires more vigorous traction and indicates Simpson’s forceps as the preferred instrument. The rotation must be performed in a wide movement of circumduction of the handles. 9 20

A second strategy for applications in posterior varieties, which has the advantage of avoiding detachment of the occiput against the perineal musculature, is to perform a wide 135° anterior rotation for the OP, followed by a single-grip extraction. This technique requires operator experience and the use of a Kielland’s forceps. Here, the slight pelvic curvature of this forceps allows the blades to be directed downwards at the time of application. Once the 135° of rotation is completed (“key through the keyhole”), the pelvic curvature of the forceps is positioned in the same direction as the maternal pelvic curvature and the cephalic detachment occurs in the OP variety, with no need for a second grip. 9 20

A third technical option for the posterior varieties, which also has the advantage of cephalic detachment in the OP variety, is to perform the 135° rotation by means of Scanzoni’s maneuver (double grasp) using a Simpson’s forceps. The technique is useful when Kielland’s forceps are not available and/or when there is an operator with dexterity and appreciation of the procedure. The first application is performed with the pelvic curvature of the forceps directed upwards, towards the fetal bregma. After a 135° rotation performed with a wide circumduction movement of the handles, the pelvic curvature of the forceps is directed downwards and the cephalic pole in the OP variety. As Simpson’s forceps blades have a wide pelvic curvature, the instrument must be removed for a second application, and extraction of the cephalic pole with the pelvic curvature of the blades facing downwards is prohibited. The second grip follows the principles for application and detachment of the fully rotated cephalic pole. 9 20

Among these three techniques in posterior presentations, the 135° rotation with Kielland’s forceps in a single grip is undoubtedly the most advantageous as it promotes detachment in the OP variety, with a reduction in vaginal manipulation and use of force. 9 20

**Figure 18.**
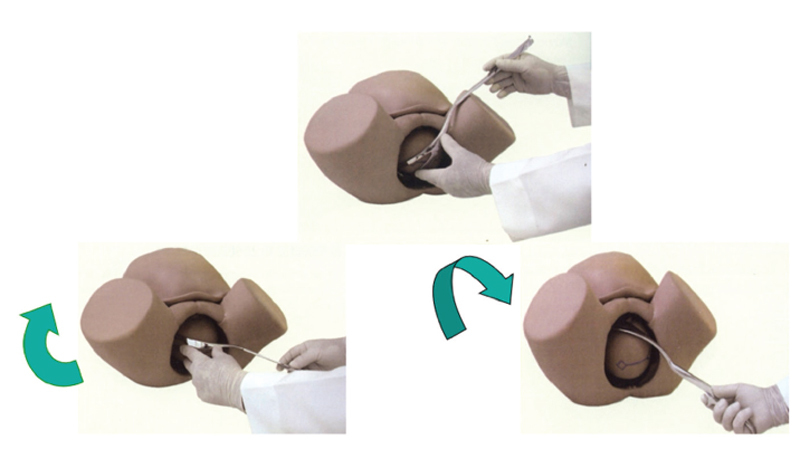
Source: Adapted from Benzecry (2006). 9 Application of the right branch of Kielland’s forceps to the anterior parietal bone by means of the Lachapelle maneuver (translation, lowering and torsion of the handle) in the left occiput transverse position

Kielland’s forceps are the most indicated for application in transverse varieties (right occiput transverse [ROT] and left occiput transverse [LOT]). The option of applying the anterior branch first is advantageous, as it requires a wide Lachapelle maneuver, which can be hampered when one chooses to apply the first branch posteriorly in the pelvis. As this displaces the cephalic pole anteriorly, insertion of the anterior branch by means of the triple spiral movement is made difficult. Thus, the first branch is applied anteriorly, through movements of translation, lowering and twisting of the handle (Lachapelle maneuver - itinerant technique) ( Figure 18 ). The second branch is introduced later, directly. Asynclitism is often present in these position varieties, requiring its correction prior to assessment of the correct grip, rotation and traction. For this, one of the branches must penetrate more than the other in the birth canal, depending on the type of asynclitism (anterior or posterior). The correction for the synclitism position is performed by sliding the already articulated branches of the forceps. It is recommended to pull the branch that penetrated the most into the birth canal, avoiding to push the branch that penetrated the least in order to avoid trauma to the upper portions of the birth canal. Correction of asynclitism is confirmed using Laufe’s criteria, before performing rotation (“key through the keyhole”) and traction. 9 20

**Figure 19.**
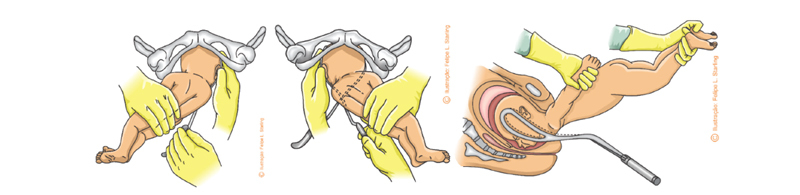
Source: Photographic record by the authors. Application of Piper’s forceps on the stuck head with the occiput positioned anteriorly (occiput posterior position)

Because it has larger branches and ample perineal curvature, Piper’s forceps are the most indicated for impaction of the head (breech baby) ( Figure 2 ). In the technique, an assistant lifts the body of the fetus by the lower limbs or with a compress positioned under the fetal abdomen. Positioned horizontally, the left branch is introduced first, directly. Subsequently, the right branch is introduced in a similar way without greater difficulty in articulating with the left branch. When assessing the correct grip, the facial line must be equidistant from the articulated branches of the forceps, the finger must not penetrate through the fenestrae of the blades and the chin must be close to or at most 1.5cm from the plane of the shanks. In the previous varieties, the application is performed in OP with the branches introduced under the fetal body. Traction should be axial, following the curvature of the maternal pelvis until the suboccipital region is positioned under the pubic arch. The head is extracted by accentuating the flexion and subsequently moving the articulated instrument towards the maternal abdomen. The instrument must be disarticulated before complete extraction of the cephalic pole ( Figure 19 ). 9

In the later varieties, the branches are introduced over the fetal body and the application takes place in the OA. Traction is exerted forward, with the mandible and fetal neck resting on the superior border of the pubic symphysis. The fetal trunk is then elevated towards the maternal abdomen. 9

## How should the sequencing of instruments and handling be done in the face of failed attempts at operative vaginal delivery?

The sequential use of forceps and vacuum is associated with increased rates of cerebral, subdural, and subarachnoid hemorrhage in newborns, as well as facial nerve and brachial plexus injuries. Severe perineal lacerations are also more common. 21 The effectiveness in resolving operative vaginal delivery is greater with forceps than with vacuum extractors. Therefore, after a failed attempt at vacuum extraction, the risks of a subsequent attempt at forceps must be weighed against the risks of a cesarean section. In contrast, in situations where the forceps attempt fails, the attempt at vacuum extraction is contraindicated, and the subsequent cesarean section must be performed. 22 Before performing the cesarean section, it is recommended to de-impact the cephalic pole by means of maneuvers or other instruments (Coyne, Sellheim or Murless levers; C-Snorkel; fetal pillow). 23

## What is the role of ultrasound in operative vaginal delivery?

Ultrasonography can be used to confirm the diagnosis of the variety of position and height of the cephalic pole, helping to assess the probabilities of success and the risks of operative vaginal delivery. It has also been described in the objective monitoring of rotational applications. The parameters evaluated when determining the position and variety of position are the cerebellum, orbits and midline falx. Ultrasonographic measurements of head circumference, the distance between the perineum and the fetal skull, and the angle of progression are predictive of difficult operative vaginal deliveries. Studies reveal that ultrasound increases the diagnostic accuracy of positional variety with no differences in maternal or neonatal outcomes. 24 Therefore, there is still not enough evidence to recommend the routine use of abdominal or perineal ultrasound for assessment of the station, flexion and descent of the fetal head in the second stage of labor. 5

## What are the recommendations for episiotomy, antibiotic prophylaxis, and thromboprophylaxis in operative vaginal delivery?

Operative vaginal delivery is one of the indications for episiotomy, which must be selective. Current recommendations do not advocate routine episiotomy in operative vaginal delivery given the poor healing and discomfort associated with mediolateral episiotomy, and the risk of injury to the anal sphincter and rectum with median episiotomy. 4 25 26 However, in the context of instrumental delivery, episiotomy is presented as a risk modifying procedure, and not as a treatment for severe perineal lacerations. The search for the best scientific evidence regarding the effect of episiotomy on the risk of severe perineal lacerations in operative vaginal delivery, to be obtained through randomized clinical trials, is hampered by the challenge of composing dichotomized groups into 0% and 100% performance of the procedure, as well as in biases introduced by the heterogeneity of operators’ skills and the difficulty in ensuring that an appropriate incision angle (between 40° and 60°) is always obtained in the intervention group. Therefore, the value of large observational studies remains, which demonstrate that mediolateral episiotomy can play an important role in preventing severe perineal lacerations during operative vaginal delivery. 27 Selecting parturients for undergoing or not an episiotomy during operative vaginal delivery requires operator experience and skill, especially when opting for posterior cephalic detachment (OA). The moment of the episiotomy should not precede the test of traction and the rotation maneuvers, avoiding the performance of the procedure in the event of a failed attempt at operative vaginal delivery. Therefore, after the descent of the presentation, with the occiput below the pubic symphysis, in the anterior detachment (OP), the elevation of the cephalic pole begins by means of the displacement of the articulated handles of the forceps towards the maternal abdomen and the evaluation of the need for episiotomy. 28 29 A single intravenous dose of antibiotics is recommended in operative vaginal delivery, as it significantly reduces the likelihood of infection and has few adverse events. Correct asepsis techniques and the use of personal protective equipment are also advised. 30 After operative vaginal delivery, puerperal women should be reassessed for the risk of venous thromboembolism and the need for thromboprophylaxis. Risk factors, such as prolonged labor and immobility are frequently associated with instrumental delivery. 31

## What are the main maternal and neonatal complications of operative vaginal delivery?

When used in the correct technique, forceps and vacuum extractors have low rates of maternal and neonatal complications. 4 5 32 Maternal complications associated with the use of forceps are lacerations in the birth canal (uterine, cervical and/or vaginal), severe perineal lacerations (third and fourth degrees), prolonged episiotomy, bladder and/or urethral injuries, and hematomas. 33 Neonatal complications associated with forceps include subgaleal hemorrhages, abrasions, facial lacerations, ocular compressions, corneal abrasions, paralysis of the facial and/or hypoglossal nerves, cervical spine injury, skull fracture, and intracranial hemorrhage. 4 5 34 35 Third- and fourth-degree (severe) perineal lacerations are also maternal complications related to vacuum extraction, but in smaller proportions than instrumental delivery with forceps. The main neonatal complications in vacuum extraction occur because the traction is applied to the scalp. The main ones are scalp lacerations, cephalohematomas and intracranial, subgaleal and retinal hemorrhages. Cephalohematomas are more frequently associated with application errors (cups attached outside the flexion point) and failures in fetal extraction. They are more likely to occur with increasing duration of vacuum extractions. 36 Even though there is association between operative vaginal delivery and severe perineal lacerations, pelvic floor function and sexual function scores within one year of delivery do not appear to differ in relation to cesarean delivery. 37 Obstetricians should be trained to recognize and treat maternal complications. Neonatologists should be informed about the technique used in operative vaginal delivery in order to assess and observe potential associated neonatal complications. 4 5

## What should analgesia and urinary tract care be like after operative vaginal delivery?

Postpartum analgesia with non-steroidal anti-inflammatory drugs and paracetamol should be routinely performed after assisted birth with forceps or a vacuum extractor. 38 Postpartum women should be instructed about the risk of urinary retention present with the association between analgesia and operative vaginal delivery. They should be encouraged to empty their bladder in the postpartum period and have their urinary time and volume (including residual volume) monitored. Intermittent or even indwelling urinary catheterization may be necessary for 24 to 48 hours. In more lasting bladder dysfunctions, urological evaluation and clean intermittent self-catheterization may be necessary. Physical therapy can be offered as a strategy to reduce the risk of urinary retention within three months of delivery. 39

## Final considerations

In the evolution of childbirth care, forceps are the resource with the greatest potential to save lives. Although vacuum extractors are more recent, they are also effective devices for assisted birth and offer the advantage of simplifying the operative technique. With adequate knowledge and skill, the cost-effectiveness and safety of instrumental vaginal delivery are favorable and endorse current guideline recommendations for operative vaginal delivery. Despite the obvious advantages, the potential of operative vaginal delivery is currently limited both by ignorance and misuse. The progressive replacement of forceps and vacuum extractors by cesarean section motivated by the lack of preparation of the new generation of obstetricians seems to introduce a real possibility of the disappearance of these instruments from the medical practice of childbirth care. The emergence of new instruments that although less effective, require less technical skill from the operator seems to be a reflection of the current inabilities of obstetricians for operative vaginal delivery. Therefore, training in these important skills must be urgently reconsidered, before this art is lost forever.

National Specialized Commission on Obstetric Emergencies of the Brazilian Federation of Gynecology and Obstetrics Associations

President:

Álvaro Luiz Lage Alves

Members:

Gabriel Costa Osanan

Samira El Maerrawi Tebecherane Haddad

Adriana Amorim Francisco

Alexandre Massao Nozaki

Brena Carvalho Pinto de Melo

Breno José Acauan Filho

Carla Betina Andreucci Polido

Eduardo Cordioli

Frederico José Amedée Peret

Gilberto Nagahama

Laíses Braga Vieira

Lucas Barbosa da Silva

Marcelo Guimarães Rodrigues

Rodrigo Dias Nunes

Roxana Knobel
